# Rapid Acute Physiology Score versus Rapid Emergency Medicine Score in Trauma Outcome Prediction; a Comparative Study

**Published:** 2017-01-10

**Authors:** Babak Nakhjavan-Shahraki, Masoud Baikpour, Mahmoud Yousefifard, Zahra Sadat Nikseresht, Samaneh Abiri, Jalaledin Mirzay Razaz, Gholamreza Faridaalaee, Mahboob Pouraghae, Sahar Shirzadegan, Mostafa Hosseini

**Affiliations:** 1 Sina Trauma and Surgery Research Center, Tehran University of Medical Sciences, Tehran, Iran.; 2 Department of Medicine, School of Medicine, Tehran University of Medical Sciences, Tehran, Iran.; 3 Physiology Research Center and Department of Physiology, Faculty of Medicine, Iran University of Medical Sciences, Tehran, Iran.; 4 Department of Emergency Medicine, Jahrom University of Medical Sciences, Jahrom, Iran.; 5 Department of Community Nutrition, Faculty of Nutrition and Food Technology, Shahid Beheshti University of Medical Sciences, Tehran, Iran.; 6 Department of Emergency Medicine, Maragheh University of Medical Sciences, Maragheh, Iran.; 7 Emergency Medicine Research Team, Tabriz University of Medical Sciences, Tabriz, Iran.; 8 Department of Epidemiology and Biostatistics, School of Public Health, Tehran University of Medical Sciences, Tehran, Iran.

**Keywords:** Multiple trauma, trauma severity indices, decision support techniques, prognosis, patient outcome assessment

## Abstract

**Introduction:**

Rapid acute physiology score (RAPS) and rapid emergency medicine score (REMS) are two physiologic models for measuring injury severity in emergency settings. The present study was designed to compare the two models in outcome prediction of trauma patients presenting to emergency department (ED).

**Methods:**

In this prospective cross-sectional study, the two models of RAPS and REMS were compared regarding prediction of mortality and poor outcome (severe disability based on Glasgow outcome scale) of trauma patients presenting to the EDs of 5 educational hospitals in Iran (Tehran, Tabriz, Urmia, Jahrom and Ilam) from May to October 2016. The discriminatory power and calibration of the models were calculated and compared using STATA 11.

**Results:**

2148 patients with the mean age of 39.50±17.27 years were studied (75.56% males). The area under the curve of REMS and RAPS in predicting in-hospital mortality were calculated to be 0.93 (95% CI: 0.92-0.95) and 0.899 (95% CI: 0.86-0.93), respectively (p=0.02). These measures were 0.92 (95% CI: 0.90-0.94) and 0.86 (95% CI: 0.83-0.90), respectively, regarding poor outcome (p=0.001). The optimum cut-off point in predicting outcome was found to be 3 for REMS model and 2 for RAPS model. The sensitivity and specificity of REMS and RAPS in the mentioned cut offs were 95.93 vs. 85.37 and 77.63 vs. 83.51, respectively, in predicting mortality. Calibration and overall performance of the two models were acceptable.

**Conclusion:**

The present study showed that adding age and level of arterial oxygen saturation to the variables included in RAPS model can increase its predictive value. Therefore, it seems that REMS could be used for predicting mortality and poor outcome of trauma patients in emergency settings.

## Introduction

Appropriate and timely management is strongly associated with a decrease in morbidity and mortality rates in trauma patients (3). Emergency physicians provide one of the main levels of care in management of trauma patients. However, the constant overcrowding of emergency departments (ED) might deprive the physicians and nurses of the time for appropriate management of patients. 

In this regard, one of the best ways to perform a quick assessment of the patients and take necessary measures accordingly is appropriate application of scoring systems (4-11). Application of screening tools to lower the time needed for assessment of patients can considerably improve the quality of care (12), increase the efficacy of treatments and lower morbidity and mortality rates. Various scoring systems have been developed and undergone gradual modifications throughout decades to increase their efficacy, accuracy and validity. Despite the improvements in these scoring systems, unfortunately they still have few shortcomings (13) and using them can be associated with multiple limitations. These limitations include the need for complicated calculations, the great number of variables they assess, and sometimes lack of validity evaluation in different clinical settings. Therefore, research in this field is still in progress and each year some new models are developed. 

In recent years, health organizations have suggested to develop a physiologic scoring system for early detection of high-risk patients in order to regulate management of trauma patients and consequently, lower the burden of trauma injuries (14). One of these scoring systems was the Rapid Acute Physiology Score (RAPS), the abbreviated version of the acute physiology and chronic health evaluation (APACHE II) score in which physiologic variables including the heart rate, blood pressure, respiratory rate and Glasgow Coma Scale (GCS) were considered as prognostic factors in trauma patients. Although the prognostic value of this model has been found to be acceptable for clinical use, researchers are still trying to improve its accuracy (19). 

The other recently presented model is Rapid Emergency Medicine Score (REMS). This model incorporates the level of arterial oxygen saturation (O_2_ sat) and chronological age of patients with the variables included in the RAPS model and was initially proposed for predicting mortality in non-surgical patients admitted to ED (19, 20). However, the validity of this model in trauma patients has been evaluated in only a few studies. 

Since a limited number of variables have been included in RAPS and REMS models, assessing and calculating the score based on them is feasible and they can be easily used in EDs. However, only a few studies have compared the two models with each other (19) and disagreements still exist on which one to use when assessing a trauma patient. Accordingly, the present study aimed to assess and compare the prognostic value of RAPS and REMS models for in-hospital mortality and poor outcome of trauma patients presenting to ED. 

## Methods

***Study design and setting***

In this cross-sectional study, the two models of RAPS and REMS were compared in predicting the in-hospital mortality and poor outcome (severe disability based on Glasgow outcome scale) in trauma patients presenting to ED. The study protocol was evaluated and approved by the Ethics Committee of Tehran University of Medical Sciences. The authors adhered to the guidelines proposed by the Declaration of Helsinki throughout the study. The patients or their family members signed an informed written consent for participating in the study. 

***Participants***

Data were gathered prospectively from EDs of 5 educational hospitals in Iran (Tehran, Tabriz, Urmia, Jahrom and Ilam) from May to October 2016. Trauma patients aged over 18 years old referring to ED were included through a convenience sampling method. Pregnant women and patients who expired at the event scene were excluded. 

***Data gathering***

In each ED, an emergency medicine physician prospectively gathered data on demographic characteristics of the patients (age, gender and trauma mechanism), their signs and symptoms and findings of their physical examination and recorded the information in data collection forms. These data included all the factors needed for calculating RAPS and REMS models (19). Gathered information included age, body temperature, systolic and diastolic blood pressures (from which the mean arterial pressure was calculated), heart rate, respiratory rate, level of oxygen saturation and the patient’s level of consciousness based on GCS. All these factors were measured for the patients on arrival and then they were followed during their admission to record their final outcome (expired vs. alive) and the condition in which they were discharged from the hospital (full recovery, moderate disability, severe disability or vegetative state). 

***Outcome measurement***

The outcome of the patients on discharge from the hospital was evaluated using Glasgow outcome scale (21). The primary outcome was in-hospital mortality and the secondary outcome was poor outcome defined as developing severe disabilities. 

***Statistical analysis***

The minimum sample size was calculated to be 1894 patients considering an in-hospital mortality rate of 5.2% among trauma patients (22), a confidence interval of 95% (α=0.05), a power of 90% (β=0.1) and a maximum error of 1.5% in estimating the mortality rate (d=0.01). 

Data were entered into SPSS software version 21.0 and were analyzed by STATA 11.0 software. All the patients had two different scores based on REMS and RAPS models. 

Area under the receiving operating characteristics curve (AUC), sensitivity, specificity, and positive and negative likelihood ratios with 95% confidence intervals (95% CI) were calculated for each model and subsequently the discriminatory power was evaluated. AUC of the two models were compared based on the method proposed by Cleves and Rock (23). 

General calibration was assessed by drawing calibration plots, in which the number of observed versus predicted mortality or poor outcome per decile of the linear predictor of RAPS or REMS models were compared. In this plot, the reference line, with an intercept of zero and a slope of one, shows perfect calibration. 

Overall performance was also evaluated by assessing the predictive reliability and predictive accuracy through calculating Brier score. Finally, the Spearman’s rank coefficient was calculated to assess the concordance between REMS-predicted and RAPS-predicted percentage of mortality and poor outcome. A p<0.05 was considered as the level of significance in all the analyses. 

## Results

***Baseline characteristics***

Data from a total of 2148 patients were gathered. The mean age of included patients was 39.50±17.27 years and 75.56% of them were male. Motorcycle accident (27.51%), Car rider accident (24.12%) and the pedestrian (17.60%) were the most common mechanisms of injury. The mean values of vital signs, level of consciousness and arterial oxygen saturation in the studied trauma patients are presented in Table 1. Patients were discharged from the hospital with a good recovery and mild disability in 75.88% of cases, moderate disability in 15.92% and severe disability in 2.47% of them. Eventually, 5.73% of the included patients expired. 

***Discrimination***

**Figure 1:**
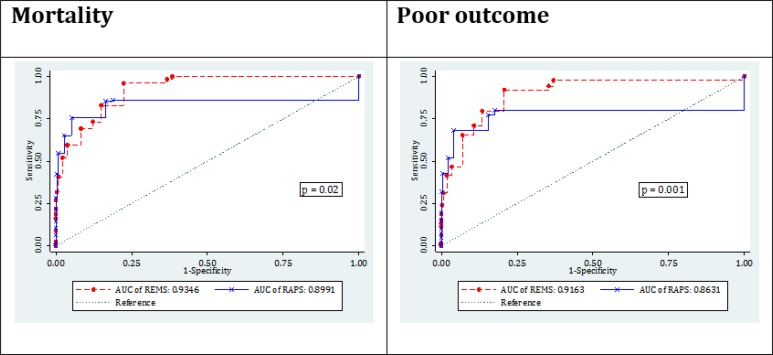
Area under the curve (AUC) of rapid emergency medicine score (REMS) and rapid acute physiology score (RAPS) in prediction of in-hospital mortality and poor outcome

Figure 1 depicts the AUC of RAPS and REMS models in predicting mortality and poor outcome. The AUC of REMS and RAPS models in predicting in-hospital mortality were 0.93 (95% CI: 0.92-0.95) and 0.899 (95% CI: 0.86-0.93), respectively, and the difference between the two was found to be statistically significant (p=0.02). Similarly, the AUC of REMS and RAPS in predicting poor outcome were calculated to be 0.92 (95% CI: 0.90-0.94) and 0.86 (95% CI: 0.83-0.90), respectively, with the differences being statistically significant (p=0.001).

The optimum cut-off value for REMS model in predicting mortality and poor outcome was 3 while this figure was found to be 2 for the RAPS model. Screening performance characteristics of REMS and RAPS models are presented in Table 2. As can be seen, the sensitivity of REMS model was considerably higher than RAPS (95.63 vs. 85.37), while its specificity was found to be lower than that of the RAPS model in predicting mortality (77.63 vs. 83.51). Similar findings were yielded for predicting poor outcome in patients. Since both of these models were developed for screening trauma patients, the model with a higher sensitivity would be more suitable for this purpose. Therefore, it seems that the value of REMS model in predicting mortality and poor outcome in trauma patients is higher than the RAPS model. 

***Calibration***

**Figure 2:**
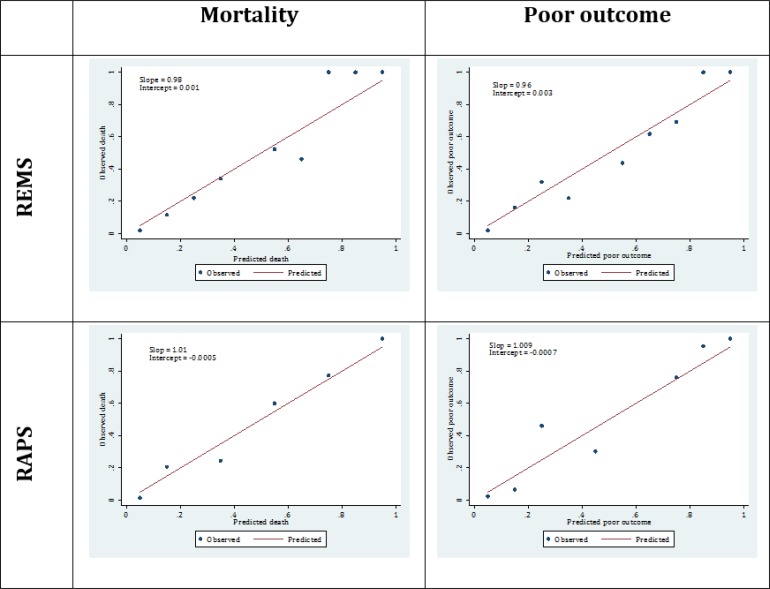
Calibration plots of rapid emergency medicine score (REMS) and rapid acute physiology score (RAPS) in prediction of in-hospital mortality and poor outcome

Figure 2 depicts the calibration plots of REMS and RAPS models, showing acceptable curves for both models in predicting mortality and poor outcome. Calibration plot of REMS model in predicting in-hospital mortality had a slope and intercept of 0.98 and 0.001, respectively, while these figures were found to be 0.96 and 0.003, respectively, for predicting poor outcome. As for the RAPS model the calibration plot for predicting mortality had a slope and intercept of 1.01 and -0.0005 while these figures were calculated to be 1.009 and -0.0007, respectively, for predicting poor outcome. These plots indicate that both models are perfect in predicting both mortality and poor outcome in trauma patients. 

***Overall performance***

Brier score for REMS model in predicting mortality was 0.034 and the scaled reliability was found to be 0.0004. For the RAPS model, these figures were calculated to be 0.028 and 0.0001, respectively. Similar results were obtained on prediction of poor outcome. These findings confirm the high predictive accuracy and reliability of the two models (**Table 3**).

**Figure 3:**
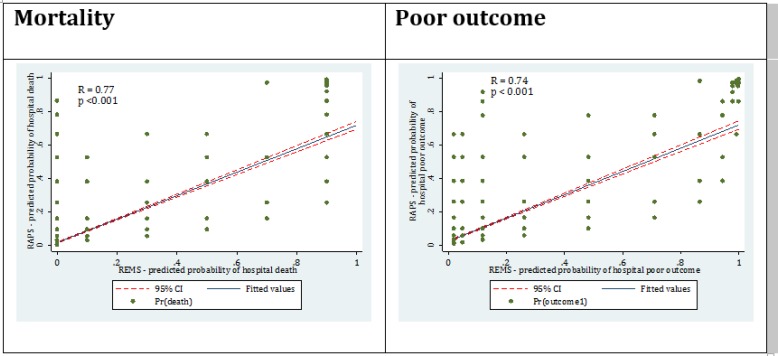
Concordance between rapid emergency medicine score (REMS) predicted and rapid acute physiology score (RAPS) predicted percentage of mortality and poor outcome

Finally, concordance between REMS and RAPS models was evaluated and a good correlation was observed in the predicted risk of mortality (r=0.77; p <0.001) and poor outcome (r=0.77; p<0.001) between the two models (Figure 3).

## Discussion

The findings of the present study showed that both REMS and RAPS models have acceptable predictive values for mortality and poor outcome of adult trauma patients referring to EDs. However, in comparison it seems that the REMS model is slightly better than the RAPS model for this purpose. 

These findings were congruent with the results of the study conducted by Olsson et al. that showed the REMS model to be a strong predictor of in-hospital mortality in patients referring to EDs and has a higher predictive value compared to RAPS model (24). These researchers aimed to assess the predictive value of REMS model in three further studies, two of which indicated that this model is a strong tool for predicting mortality in non-surgical patients (19, 20). The third study showed that even with incorporation of the Charlson comorbidity index in the analyses, the REMS model has a high predictive value for mortality of non-surgical patients (25). In another study conducted on 3680 patients, Imholff et al. showed that a higher REMS score is associated with an increase in the mortality rate of trauma patients. These authors suggest that this scoring system is a simple and accurate predictor for in-hospital mortality of trauma patients (22). In their survey aiming to evaluate the role of REMS model in predicting mortality of patients infected with Vibrio vulnificus, Kuo et al. also found that this model provides an acceptable predictive value for mortality of patients (26). 

Ha et al. aimed to compare the prognostic performance of the two REMS model and Worthing Physiological Scoring system in predicting mortality of patients referring to EDs and found that both models have acceptable prognostic performances, with the Worthing Physiological Scoring system being slightly better that the REMS model (27). Bulut et al. evaluated 2000 patients and reported that although both models have moderate predictive values, but the prognostic value of REMS model for mortality of patients referring to EDs was significantly higher than Modified Early Warning Score (28). As can be seen, slight disagreements can be observed between the results of various studies considering the prognostic value of REMS and RAPS models for mortality of patients, which can be attributed to the differences in settings of the surveys.

**Table 1:** Baseline characteristics of studied patients

Variable	Value
**Age (year)**	39.50 ± 17.27
**Gender (n, %)**	
Male	1623 (75.56)
Female	525 (24.44)
**Mechanism of trauma**	
Motorcycle accident	591 (27.51)
Car rider accident	518 (24.12)
Pedestrian	378 (17.60)
Falls more than 3 meters	152 (7.08)
Falls less than 3 meters	201 (9.36)
Other	308 (14.34)
**GCS**	14.4 ± 2.19
**HR (beat/minute)**	87.60 ± 15.63
**SBP (mmHg)**	115.38 ± 15.36
**DBP (mmHg)**	73.49 ± 10.07
**O2 sat (%)**	94.78 ± 5.80
**Temperature (Celsius)**	36.81 ± 0.90
**RR (number/minute)**	16.46 ± 6.15
**Outcome **	
Good recovery	1630 (75.88)
Moderate disability	342 (15.92)
Severe disability	53 (2.47)
Death	123 (5.73)

Data were presented as mean ± standard deviation or frequency and percentage; GCS: Glasgow coma scale; HR: heart rate; SBP: systolic blood pressure; DBP: diastolic blood pressure; O_2_ Sat: arterial oxygen saturation; RR: respiratory rate.

**Table 2:** Screening performance characteristics of rapid emergency medicine score (REMS) and rapid acute physiology score (RAPS) in prediction of mortality and poor outcome

**Characteristics**	**Mortality**		**Poor outcome**	
	**REMS**	**RAPS**	**REMS**	**RAPS**
**True positive**	118	105	162	136
**True negative**	1572	1691	1563	1669
**False positive**	453	334	409	303
**False negative**	5	18	14	40
**Sensitivity**	95.93 (90.30-98.49)	85.37 (77.59-90.86)	92.04 (86.75-95.42)	77.27 (70.23-83.09)
**Specificity**	77.63 (75.74-79.42)	83.51 (81.80-85.08)	79.26 (77.39-81.02)	84.64 (82.95-86.18)
**Positive LR**	4.29 (3.92-4.69)	5.18 (4.58-5.85)	4.44 (4.03-4.89)	5.03 (4.41-5.72)
**Negative LR**	0.05 (0.02-0.12)	0.18 (0.11-0.27)	0.10 (0.06-0.17)	0.27 (0.20-0.35)

*, Data are presented as estimated value and 95% confidence interval. LR: Likelihood ratio

**Table 3:** Overall performance of rapid emergency medicine score (REMS) and rapid acute physiology score (RAPS) in prediction of in-hospital mortality and poor outcome

**Characteristics**	**Mortality**		**Poor outcome**	
	**REMS**	**RAPS**	**REMS**	**RAPS**
**Brier score**	0.034	0.028	0.049	0.043
**Scaled reliability**	0.0004	0.0001	0.0005	0.0003

Various scoring systems have been developed for classification of injuries, which include physiologic and anatomic systems, specialized trauma scoring systems and combined scores (29). Each of these systems has their own specific limitations and advantages, but a scoring system that is going to be used in the emergency settings should involve fewer variables and be easy to use. In this regard, the RAPS model, which includes few variables, might be a good candidate for application in emergency settings, but to increase its predictive value, the two variables of age and arterial oxygen saturation level were added to the model and the REMS model was developed. Results of the present study, based on calculated AUCs, showed that predictive value of RAPS model for in-hospital mortality (AUC=0.899) and poor outcome of patients (AUC=0.86) were good, while the prognostic values of REMS model were found to be excellent for mortality (AUC=0.93) and poor outcome (p=0.92). 

The relatively large sample population and the multi-center setting can be considered as the strengths of the present study, which warrants its power. Having included patients from five cities of Tehran, Tabriz, Urmia, Jahrom and Ilam reassured the representativeness of the findings to the whole Iranian population. Accordingly, it seems that REMS model has a higher value for predicting in-hospital mortality and poor outcome of trauma patients presenting to EDs. 

## Limitations

Employing a convenience sampling method suggests presence of selection bias in this study. Another limitation of this survey was inclusion of body temperature in the analyses based on an axillary reading which might not be accurate particularly in overcrowded emergency settings and can affect the final interpretation of results. 

## Conclusion:

The present study showed that adding age and the level of arterial oxygen saturation to the variables included in the RAPS model can increase its predictive value. Therefore, it seems that REMS could be used for predicting mortality and poor outcome of trauma patients in emergency settings. 

## References

[1] Meheš M, Abdullah F (2011). Global Surgery and Public Health: A New Paradigm. Archives of Surgery.

[2] Mathers CD, Loncar D (2006). Projections of Global Mortality and Burden of Disease from 2002 to 2030. PLoS Med.

[3] Shortell SM, Zimmerman JE, Rousseau DM, Gillies RR, Wagner DP, Draper EA The performance of intensive care units: does good management make a difference?. Medical care.

[4] Hosseini M, Ghelichkhani P, Baikpour M, Tafakhori A, Asady H, Ghanbari MJH (2015). Diagnostic Accuracy of Ultrasonography and Radiography in Detection of Pulmonary Contusion; a Systematic Review and Meta-Analysis. Emergency.

[5] Rahimi-Movaghar V, Yousefifard M, Ghelichkhani P, Baikpour M, Tafakhori A, Asady H (2016). Application of Ultrasonography and Radiography in Detection of Hemothorax; a Systematic Review and Meta-Analysis. Emergency.

[6] Rahimi-Movaghar V, Yousefifard M, Ghelichkhani P, Baikpour M, Tafakhori A, Asady H (2016). Application of Ultrasonography and Radiography in Detection of Hemothorax: a Systematic Review and Meta-Analysis. EMERGENCY-An Academic Emergency Medicine Journal.

[7] Safari S, Yousefifard M, Baikpour M, Rahimi-Movaghar V, Abiri S, Falaki M (2016). Validation of thoracic injury rule out criteria as a decision instrument for screening of chest radiography in blunt thoracic trauma. Journal of clinical orthopaedics and trauma.

[8] Safari S, Yousefifard M, Hashemi B, Baratloo A, Forouzanfar MM, Rahmati F (2016). The value of serum creatine kinase in predicting the risk of rhabdomyolysis-induced acute kidney injury: a systematic review and meta-analysis. Clinical and experimental nephrology.

[9] Yousefifard M, Baikpour M, Ghelichkhani P, Asady H, Darafarin A, Amini Esfahani MR (2016). Comparison of Ultrasonography and Radiography in Detection of Thoracic Bone Fractures; a Systematic Review and Meta-Analysis. Emergency.

[10] Yousefifard M, Baikpour M, Ghelichkhani P, Asady H, Darafarin A, Esfahani MRA (2016). Comparison of Ultrasonography and Radiography in Detection of Thoracic Bone Fractures; a Systematic Review and Meta-Analysis. Emergency.

[11] Yousefifard M, Baikpour M, Ghelichkhani P, Asady H, Nia KS, Jafari AM (2015). Screening Performance Characteristic of Ultrasonography and Radiography in Detection of Pleural Effusion; a Meta-Analysis. Emergency.

[12] de Souza Nogueira L, de Alencar Domingues C, Poggetti RS, de Sousa RMC (2014). Nursing workload in intensive care unit trauma patients: analysis of associated factors. PloS one.

[13] Staff T, Eken T, Wik L, Røislien J, Søvik S (2013). Physiologic, demographic and mechanistic factors predicting New Injury Severity Score (NISS) in motor vehicle accident victims. Injury.

[14] Kumagai G, Tsoulfas P, Toh S, McNiece I, Bramlett HM, Dietrich WD (2013). Genetically modified mesenchymal stem cells (MSCs) promote axonal regeneration and prevent hypersensitivity after spinal cord injury. Experimental neurology.

[15] Morgan R, Williams F, Wright M (1997). An early warning scoring system for detecting developing critical illness. Clin Intensive Care.

[16] de Pennington J, Laurenson J, Lebus C, Sihota S, Smith P (2005). Evaluation of early warning systems on a medical admissions unit. Journal of the Intensive Care Society.

[17] Goldhill D (2005). Preventing surgical deaths: critical care and intensive care outreach services in the postoperative period. British journal of anaesthesia.

[18] Williams E, Subbe C, Gemmell L, Morgan R, Park G, McElligot M (2003). Outreach critical care—cash for no questions?. British journal of anaesthesia.

[19] Olsson T, Lind L (2003). Comparison of the Rapid Emergency Medicine Score and APACHE II in Nonsurgical Emergency Department Patients. Academic Emergency Medicine.

[20] Olsson T, Terent A, Lind L (2004). Rapid Emergency Medicine Score Can Predict Long-term Mortality in Nonsurgical Emergency Department Patients. Academic Emergency Medicine.

[21] Anderson SI, Housley AM, Jones PA, Slattery J, Miller JD (1993). Glasgow Outcome Scale: an inter-rater reliability study. Brain Injury.

[22] Imhoff BF, Thompson NJ, Hastings MA, Nazir N, Moncure M, Cannon CM (2014). Rapid Emergency Medicine Score (REMS) in the trauma population: a retrospective study. BMJ Open.

[23] Cleves MA, Rock L (2002). From the help desk: Comparing areas under receiver operating characteristic curves from two or more probit or logit models. The Stata Journal.

[24] Olsson T, Terént A, Lind L (2004). Rapid Emergency Medicine Score: a new prognostic tool for in‐hospital mortality in nonsurgical emergency department patients. Journal of internal medicine.

[25] Olsson T, Terent A, Lind L (2005). Charlson Comorbidity Index can add prognostic information to Rapid Emergency Medicine Score as a predictor of long-term mortality. European Journal of Emergency Medicine.

[26] Kuo S-H, Tsai C-F, Li C-R, Tsai S-J, Chao W-N, Chan K-S (2013). Rapid Emergency Medicine Score as a main predictor of mortality in Vibrio vulnificus–related patients. The American Journal of Emergency Medicine.

[27] Ha DT, Dang TQ, Tran NV, Vo NY, Nguyen ND, Nguyen TV (2015). Prognostic performance of the Rapid Emergency Medicine Score (REMS) and Worthing Physiological Scoring system (WPS) in emergency department. International journal of emergency medicine.

[28] Bulut M, Cebicci H, Sigirli D, Sak A, Durmus O, Top AA The comparison of modified early warning score with rapid emergency medicine score: a prospective multicentre observational cohort study on medical and surgical patients presenting to emergency department. Emergency Medicine Journal.

[29] Lefering R (2012). Trauma scoring systems. Current opinion in critical care.

